# Towards high-resolution fat-suppressed T1-mapping of atrial fibrosis in the left atrium: a fit-free three-point method

**DOI:** 10.1186/1532-429X-17-S1-W17

**Published:** 2015-02-03

**Authors:** Dana C Peters, Stephanie L Thorn, Alda Bregazi, Christi Hawley, Mitchel R Stacy, Albert J Sinusas

**Affiliations:** 1 Diagnostic Radiology, Yale School of Medicine, New Haven, CT, USA; 2 Internal Medicine, Yale School of Medicine, New Haven, CT, USA

## Background

Atrial fibrosis identification by late gadolinium enhancement (LGE) CMR is important as a precursor to atrial fibrillation, and may impact the outcome of catheter ablation. However, the LGE enhancement in the thin atrial wall is difficult to accurately and reproducibly detect. We sought to improve identification of fibrosis through T1-mapping, generating an index of the extracellular volume fraction (ECV). In order to achieve high spatial resolution mapping for a narrow range of relevant T1-values (250-500ms) in a feasible scan time, we applied fit-free T1-mapping with only 3 TI values (3-pt). Preliminary data measuring the ECV of normal myocardium and the aortic valves—a thin fibrotic structure-- are presented.

## Methods

T1-mapping employed a NAV-gated, ECG-gated, fat-suppressed 3D multi-TI gradient echo sequence [1]. The method requires 2 RR per inversion and employed TR/TE/Θ=4.0 ms/1.7ms/15deg, 30-40 segments per RR in ventricular diastole, 30 x24 cm FOV, 1.5 x 1.5 x 3 mm^3^ true spatial resolution, ~12 minutes total scan time for measurement with three TIs, assuming 50% NAV efficiency. The T1-mapping method was targeted to post-contrast T1 values (200ms-500ms), with three inversion times: TI_1_=100ms, TI_2_=400ms, and TI_3_= ∞ (i.e. no inversion pulse), corresponding to signal intensities S_1_, S_2_ and S_∞_. The de-rectified signals were used to calculate T1 analytically: If S(TI)=A+B·exp(-TI/T1) then A=S_∞_ and T1=ΔTI /log[(S_1_-A)/(S_2_ -A)]. Therefore, no fitting or correction factor are needed.

All imaging was performed on a Siemens 1.5T (Erlangen, Germany). Phantoms with a range of T1s (200-650ms), with T1 measured by spin echo inversion recovery (IR), were imaged to compare the performance of our standard T1-mapping with 6 TIs (100 to 500ms, ∞) estimated using least-squares fitting, to the fit-free 3-pt method. T1-maps of the left atrium were also obtained in a study of Yorkshire swine (n = 5) one to two weeks after myocardial infarction, ~30 minutes post injection of 0.2mmol/kg gadobutrol. Pre-contrast TIs from literature were used.

## Results

**Figure 1:**
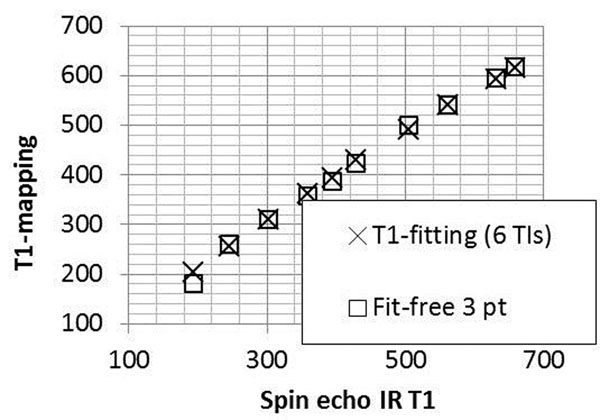
Comparison of T1 values of the fit-free 3-pt method and 6-pt method vs. gold standard (spin echo).

**Figure 2:**
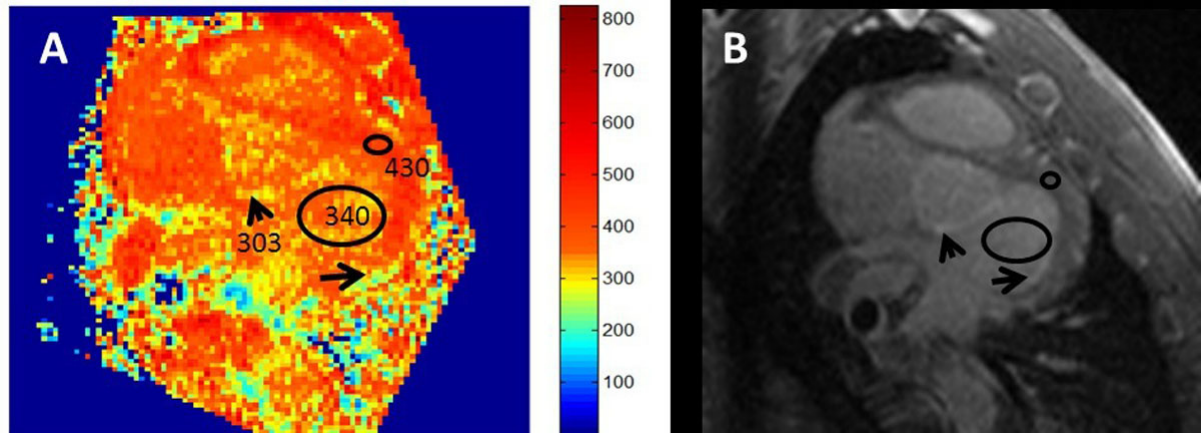
A) T1 map of the left atrium, showing lower T1 in the valves (arrow head), which are known fibrotic structures, and in a section of the posterior LA wall (arrow). B) The TI=400ms image highlights the identical areas. Using, pre-contrast T1s from literature, the visible LV myocardium had an estimated ECV of 29%, and the valvular region had an ECV of 52%, which are reasonable values for normal, and partially fibrotic structures.

Figure 1 compares the gold-standard T1s to the fit-free 3-pt method and the 6-pt method. The Bland-Altman analysis found a bias and 2SDs for the fit-free 3-pt values of 11±35ms, as compared with 3 ±17ms for the 6-pt method. Figure 2 shows T1-mapping in atria of a swine. The average ECV values for normal myocardium (26±3%) and the partially fibrotic valves (49±6%) were within the expected range.

## Conclusions

The 3-pt fit-free method is accurate for a narrow range of T1s, in a feasible but still lengthy scan time. Future work will investigate optimization of the TI choices [2]. However, we present a step towards high resolution T1-mapping for detection of atrial fibrosis.

## Funding

The authors acknowledge funding from NHLBI R01HL113352 and 1R21HL103463.
